# Young Children Selectively Imitate Models Conforming to Social Norms

**DOI:** 10.3389/fpsyg.2019.01399

**Published:** 2019-06-26

**Authors:** Katalin Oláh, Ildikó Király

**Affiliations:** ^1^ Department of Cognitive Psychology, MTA-ELTE Momentum Social Minds Research Group, Eötvös Loránd University, Budapest, Hungary; ^2^ Psychobiological Research Group, Institute of Cognitive Neuroscience and Psychology, Hungarian Academy of Sciences, Budapest, Hungary; ^3^ Cognitive Development Center, Central European University, Budapest, Hungary

**Keywords:** social learning, conventionality, norms, social categorization, imitation

## Abstract

This study investigated whether toddlers would selectively imitate a demonstrator who exhibits familiarity with cultural practices in their tool-using habits over a demonstrator who consistently uses tools in an unconventional way. Three-year-old children (*n* = 45) watched videos depicting two models, one of whom performed tool-using actions in a conventional way, while the other model deviated from social conventions. Then, both models introduced a technique to build a tower (differing in one element). Moreover, the context of the demonstration was also manipulated: in one condition, the models expressed their teaching intentions, while in the other they performed the actions without communicative signals. Children were more willing to copy the actions of the conventionally behaving model, irrespective of the context of the demonstration.

## Introduction

Human social learning is characterized by two – seemingly contradictory – important features. First, knowledge transmission should happen without applying much modification to the content of the information to ensure that behavioral patterns that provide the foundations of society stabilize, even when the function of certain actions are causally opaque to the observer (see Csibra and Gergely, 2009). For example, this makes it possible for children to rapidly learn that pushing the light switch will create light without actually understanding the underlying mechanisms. Second, novices should be able to filter out irrelevant information from the excess of stimuli reaching them at every moment (e.g., understanding that swiping dirt from the light switch before pushing it is not linked to the goal of creating light). On the large scale, the fine balance between faithful imitation and deviations from demonstrated actions contributes to humans’ ability to introduce innovations to an otherwise stable and rich body of cultural knowledge (Legare and Nielsen, 2015).

Young children seem to rely on a number of cues that may distinguish relevant pieces of information and behavioral patterns from irrelevant ones. For example, children from a very early age take into account the intentions underlying action demonstration from potential teachers. Thus, children will not reproduce actions that are merely accidental (Carpenter et al., 1998) and already infants possess a special sensitivity to detect the teaching intentions of others, helping them to identify those elements of an episode that are worth learning (as described in Natural Pedagogy theory: Csibra and Gergely, 2006, 2009). Empirical evidence lend support to the claim about the existence of an innate system making humans adept at detecting so-called ostensive-referential signals that highlight an episode as pedagogical (e.g., Senju and Csibra, 2008; Topál et al., 2008; Yoon et al., 2008; Futó et al., 2010). A number of studies have also shown that this sensitivity guides imitative behavior in children: unusual actions will be less likely to be copied if the action demonstration is not preceded by communicative signals (Király et al., 2013). Moreover, preschoolers expect information presented in a communicative context to be generalizable (Butler and Markman, 2012).

However, children from an early age may use other cues than pedagogical context to determine the relevance of information. This is exhibited, for example, in 14-month-old infants’ tendency to imitate “rationally” – that is, evaluating different aspects of the modeled behavior and its context to determine which parts of the modeled behavior should be copied (Gergely et al., 2002). At 18 months of age, children have been shown to base their judgments on which element of an action is most relevant on the type of information they received about the goal of the action beforehand (Southgate et al., 2009). In a study conducted with preschoolers, Henderson et al. (2013) have shown that 4-year-olds were more likely to learn word-referent associations when the referent was described as something purchased nearby as opposed to far-away. While these studies target slightly different questions, they highlight the fact that children from a young age are not blind imitators but, in fact, aim to extract the most relevant piece of information to obtain from a learning scenario.

In addition to selection processes that target the content of the transmission process, effective social learning should also be supported by mechanisms that help novices to make judgments about the reliability of the source of information as well. Such mechanisms should guide selection between *potential informants*. A number of studies confirm the notion that children already from a young age are discriminative in who they accept information from. For example, 3-to-5-year-old children are more willing to endorse object labels provided by a familiar individual than an unfamiliar one (Corriveau and Harris, 2009a,b); however, this initial trust evoked by familiarity is overridden by cues of accuracy (in labeling familiar objects) for 4- and 5-year-olds. Several other studies have also highlighted the importance of the past accuracy of the informant in guiding children’s learning processes (e.g., Koenig et al., 2004; Koenig and Harris, 2005; Pasquini et al., 2007). Moreover, children also retain these impressions of reliability and continue to prefer an accurate individual as information source 1 week after the first exposure to the potential informants (Corriveau and Harris, 2009a,b). On a similar vein, 14-month-old infants have been shown to monitor reliability in emotional expression and selectively imitate models that have proved reliable in this respect (Poulin-Dubois et al., 2011). At the same age, infants attend to cues of confidence and appropriate usage of tools in deciding whom to imitate (Zmyj et al., 2010). Fourteen-month-old infants are also more willing to copy novel instrumental actions performed by adults than by children (Jaswal and Neely, 2006; Zmyj et al., 2012).

Taken together, the results described above suggest that young children pay attention to cues that provide information about the knowledgeability of potential teachers in order to selectively endorse information that is most likely to be useful and appropriate. However, adaptive social learning mechanisms in humans also have to answer the challenge that lies in the diverse nature of cultural practices and adaptive behavioral patterns. Cumulative cultural evolution has led to significant variations among social groups in the scope of adaptive behavioral patterns that support survival in a particular environment (e.g., what tools we use for eating, what language we use to communicate with each other, how to use communicative gestures, which side of the road we drive on, etc.). These specific behavioral patterns and knowledge have to be transmitted through generations with the help of adaptive social learning mechanisms (Boyd and Richerson, 1996; Henrich and McElreath, 2003). We argue that under such circumstances, one challenge novices are faced with during the transmission process is that potential information sources may be reliable in one context but not in the other. Novices should not only favor informants that are confident or experienced in general but also possess knowledge that is valid in the specific social environment they grow up in. In other words, novices should be prepared to selectively endorse information coming from members of their own social group (“in-groups”). A handful of studies have already confirmed that young children indeed show selectivity based on group membership. Buttelmann et al. (2013) have shown that infants as young as 14 months old selectively imitate linguistic in-group members over people speaking in a foreign language. Howard et al. (2015) reported similar findings with 19-month-old and 3-year-old children with the constraint that the younger age group only showed selectivity when the potential informants were presented on video. We propose that language cues are effective in guiding learning processes and serve as a salient cue for social categorization because they provide direct evidence about whether the informant shares cultural knowledge with the child and thus is capable of transmitting information that is valid in their social environment.

However, language may not be the only cue that informs novices about the cultural knowledgeability of the potential teacher. Language is a reliable marker as humans possess an innate sensitivity and preference to speech (Vouloumanos and Werker, 2007) and an early-developing ability to detect subtle discrepancies in it (Nazzi et al., 1998). Moreover, these discrepancies reliably signal the boundaries of both broader (foreign language) and narrower (foreign accent) social categories. Nevertheless, we claim that language is merely one possible – though strong – cue to possessing knowledge that is specific to the child’s social group. To test this idea, this study explores whether other possible markers of cultural knowledgeability would produce convergent results in an imitation paradigm. Our candidate marker is tool-using habits as humans already from early childhood have a special (“teleofunctional”) stance toward artifact functions (Casler and Kelemen, 2007; Kelemen and Carey, 2007) that make fast and efficient learning about tool-functions possible (Casler and Kelemen, 2005) and result in viewing artifact functions as normative (Casler et al., 2009). Importantly, preschoolers expect that the same tool should not be used for multiple purposes (Casler and Kelemen, 2005). Moreover, it has been shown that children form similar representations based on the language a person speaks and the level of conventionality they exhibit in their tool-using behavior (Oláh et al., 2014). Thus, we investigated whether children would selectively imitate a model whose tool-using habits conform to the cultural norms over someone who violates the cultural norms. Our study targets one specific form of learning: copying behavioral patterns. While this may not be representative of every learning act, this is especially relevant for acquiring knowledge of cultural practices that are usually arbitrary and functionally opaque (e.g., waving to greet someone, nodding to express agreement, etc.). We tested 3-year-old children as this is the age where selective imitation based on linguistic group membership has been robustly demonstrated (Howard et al., 2015). Moreover, by this age, children already form expectations about the normative function of objects (Casler et al., 2009).

In addition, we also tested how ostensive communication would modulate any potential effect of the model’s group membership. As described above, according to the theory of Natural Pedagogy (Csibra and Gergely, 2009), an innate sensitivity to ostensive-communicative signals foster the transmission of culturally relevant knowledge in humans by pointing out the to-be-acquired information. Thus, communicative signals and the different qualities of the teacher both serve to help children acquire culturally relevant knowledge; however, little is known about how these cues interact with each other in forming the behavior of children. One possibility is that children only attend to the communicative intentions of others if the person has been proven to be a reliable source of information. In this case, children would be equally (un)willing to imitate an unconventionally behaving model following a communicative and a non-communicative action demonstration, but they would show increased motivation to copy the actions of a conventionally behaving model after a communicative demonstration. The second possibility is that children’s tendency to accept knowledge in a communicative setting is so strong that it overwrites the significance of cues of familiarity with cultural practices. In this case, children would imitate a model that gives ostensive signals irrespective of past behavior and would only differentiate based on it in the absence of such cues.

To investigate the interplay of these two factors, we presented children with videos introducing two models, one of whom performed conventional tool-using actions, while the other used the same tools in an unconventional way. After that, both models demonstrated how to build a tower from building blocks either in the presence of non-verbal communicative cues or in a completely non-communicative context. The two demonstrations varied in one element and we analyzed whether children would be more willing to copy the variant introduced by the conventionally behaving model. Importantly, both models either expressed their intention to teach (Communicative condition) or did not give any evidence of it (Non-communicative condition) and there were no conditions where the behavior of the models differed in this respect. This ensured that our two factors of interest (conventionality of behavior and communicativeness) were manipulated independently, and thus, the design would allow us to draw inferences whether one of the factors would have the power to overshadow the significance of the other.

## Materials and Methods

Ethical approval for the study was obtained from the Research Ethics Committee of the Faculty of Education and Psychology of Eötvös Loránd University (Ref. no. 2014/127).

### Participants

Fifty 3-year-old children participated in the study (mean: 39.3 months; SD: 2 months; range: 34–43 months). Children were either tested in one of two kindergartens (*n* = 38) or in the baby laboratory (*n* = 12). All children were monolingual. Five children had to be excluded from the ostensive condition due to passivity (1), having a distracting toy in their hand during testing (1), touching the apparatus too early (1), or not paying attention to the demonstration videos (2). The final sample consisted of 21 children in the ostensive condition and 24 children in the Non-ostensive condition.

### Materials

**Figure 1:**
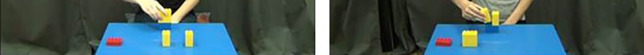
Two possible ways to build the tower (using two single blocks or one double block to build the middle section) performed by the two protagonists.

For the familiarization phase, two sets of videos were recorded of two protagonists. The videos depicted simple tool-using actions based on the stimuli developed for the study of Oláh et al. (2014). Each protagonist demonstrated two different tool-using actions either in a conventional way (cutting up a piece of paper using a pair of scissors and having a bite of food with a fork) or in an unconventional way (cutting up a banana using a pair of scissors and combing one’s hair with a fork). Each demonstration video was recorded with both protagonists in both manners. In addition, test videos were recorded with the protagonists that also had two different versions. The demonstrated action was a tower building technique, where the protagonist showed how to build a tower from three (or four) building blocks: a blue building block that was used as the base, a yellow middle section, and a red top. Crucially, the middle section could be built either from a single double block or by adding two single blocks (see Figure 1). All test videos had an ostensive-communicative and a non-communicative version, and all versions were recorded with both participants. In the ostensive videos, the protagonist started the demonstration with looking into a camera, waving and saying “Hi.” She finished the action by looking back up into the camera. In the non-ostensive videos, she simply started the demonstration with reaching for the first building block and did not look back at the camera in the end. While these manipulations seem subtle, they have been shown to effectively alter how young children process subsequent information (e.g., Senju and Csibra, 2008; Yoon et al., 2008).

For the imitation phase, the tools used in the test videos were presented for the children. Namely, the blue building block (the base of the tower), the red building block (the top of the tower), and the elements that could potentially be used for the middle section: the double yellow building block and the two single yellow blocks.

### Procedure

Children were tested individually either in a quiet room of the kindergarten or the laboratory. After escorting the child into the testing area, the experimenter told the participant that they would be watching short movies of two girls and that they should pay close attention to what happens. After that, the experimenter played the familiarization and the test videos. Each participant saw one of the protagonists perform both of the familiarization actions in a conventional way, while the other protagonist performed both actions in an unconventional way. With this, we wanted to create the impression that one protagonist consistently behaves according to social norms, while the other consistently deviates from them. Importantly, their actions were always performed in a confident way and were efficient in bringing about the desired goal. Children first watched the “fork action” being performed by both protagonists and then saw the second action (“scissors action”) being performed by the two models in the order they had appeared in the first pair of videos. After the familiarization videos, the two test videos immediately followed. Children saw one of the participants perform the tower building action with constructing the middle section from two pieces and the other protagonist building the middle part from one piece. Both participants performed the test action either in an ostensive way (Ostensive condition) or in a non-ostensive way (Non-ostensive condition). The following factors were counterbalanced across conditions: identity of the conventionally behaving model, the variant of the building technique performed by the conventionally behaving model, and the order of appearance of the two models.

### Coding

We coded whether children would choose to build the middle section from one block or two. Since children introduced significant variations into the building procedure, the following criterion was used: if children took either the double building block or the two separate pieces and placed them on the building block serving as base, then this was considered a clear choice irrespective of how they continued the building (in many cases, children ended up using up all the building blocks to build an even higher tower). If this element was missing or was performed in a completely different way (e.g., putting the two separate blocks on top of each other), then the behavior was coded as an alternative solution. A second coder coded 30% of the videos. Reliability was satisfactory (Cohen’s *κ* = 0.78).

## Results

Statistical analyses were performed in SPSS 20.0. Our main question was whether children would be more willing to imitate a conventionally behaving model than a person violating the cultural norms. Therefore, our dependent variable was which model children imitated. After coding the videos, we observed that a large proportion of participants came up with an alternative solution. For this reason, first we analyzed all the data, including children with alternative solutions. In this analysis, we used a dependent variable with three possible values (imitating the conventional model/imitating the unconventional model and alternative solution). Additionally, we performed an analysis that included only children with clear choices (children following one protagonist).

To test the effects of condition on children’s choices between the conventionally and unconventionally behaving model, we first conducted regression analyses (binary logistic and multinomial logistic for the analyses excluding and including alternative solutions, respectively) with choice of model as the dependent variable and condition (ostensive-non-ostensive), identity of the conventional model, order of presentation of the models, variant performed by the conventional model, testing location, age, and sex as predictor variables. Since none of the predictor effects reached significance (all *p* > 0.76 for the analyses excluding alternative solutions and all *p* > 0.904 for the analyses including alternative solutions), we restricted the analyses to the factors of interest (choice of model and ostensiveness of the demonstration) for our research question and used chi-square tests. Additionally, we conducted tests of distribution to explore whether children were generally more inclined to imitate the conventionally behaving model (Kolmogorov-Smirnov for the variable with three values and binomial test for the one with two values).

### Analyses Including All Participants

**Figure 2:**
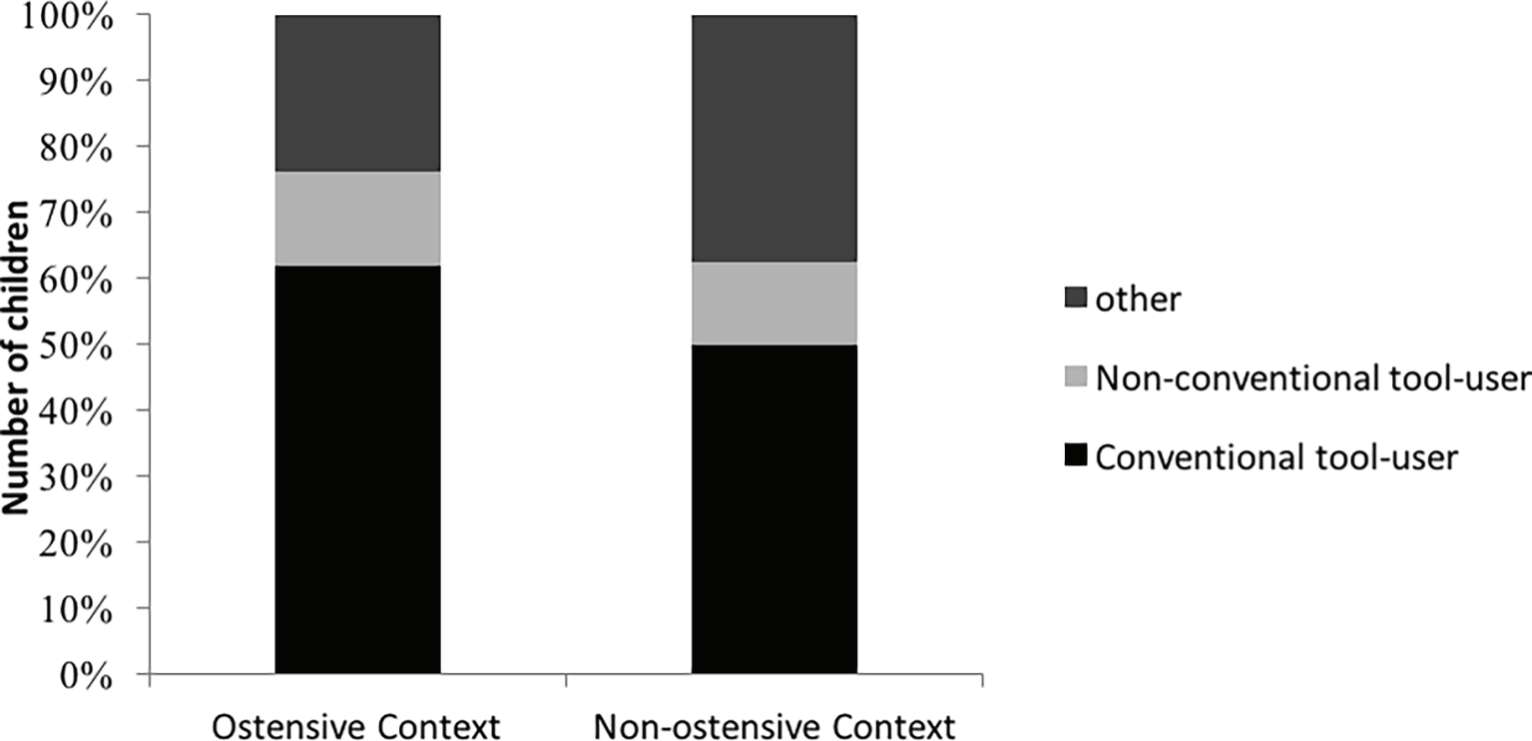
Number of children imitating the variants introduced by the two models or opting for an alternative solution in the ostensive and the non-ostensive conditions.

The results of the chi-square tests show that there was no difference between conditions in the number of children choosing to follow either of the models or opting for an alternative solution [*χ*^2^(2) = 0.277; *p* = 0.87]. The results show that more than half of the children in both conditions imitated the variant introduced by the conventionally behaving model with around the same number of participants choosing to copy the unconventionally behaving model and to come up with a new method of tower building (see Figure 2). Analyzing the distribution of behavior types across conditions, we found a significant difference between the different response types (*Z* = 2.311; *p* < 0.001), showing that participants performed the variant they had seen from the conventionally behaving model most often.

### Analyses Excluding Alternative Solutions

**Figure 3:**
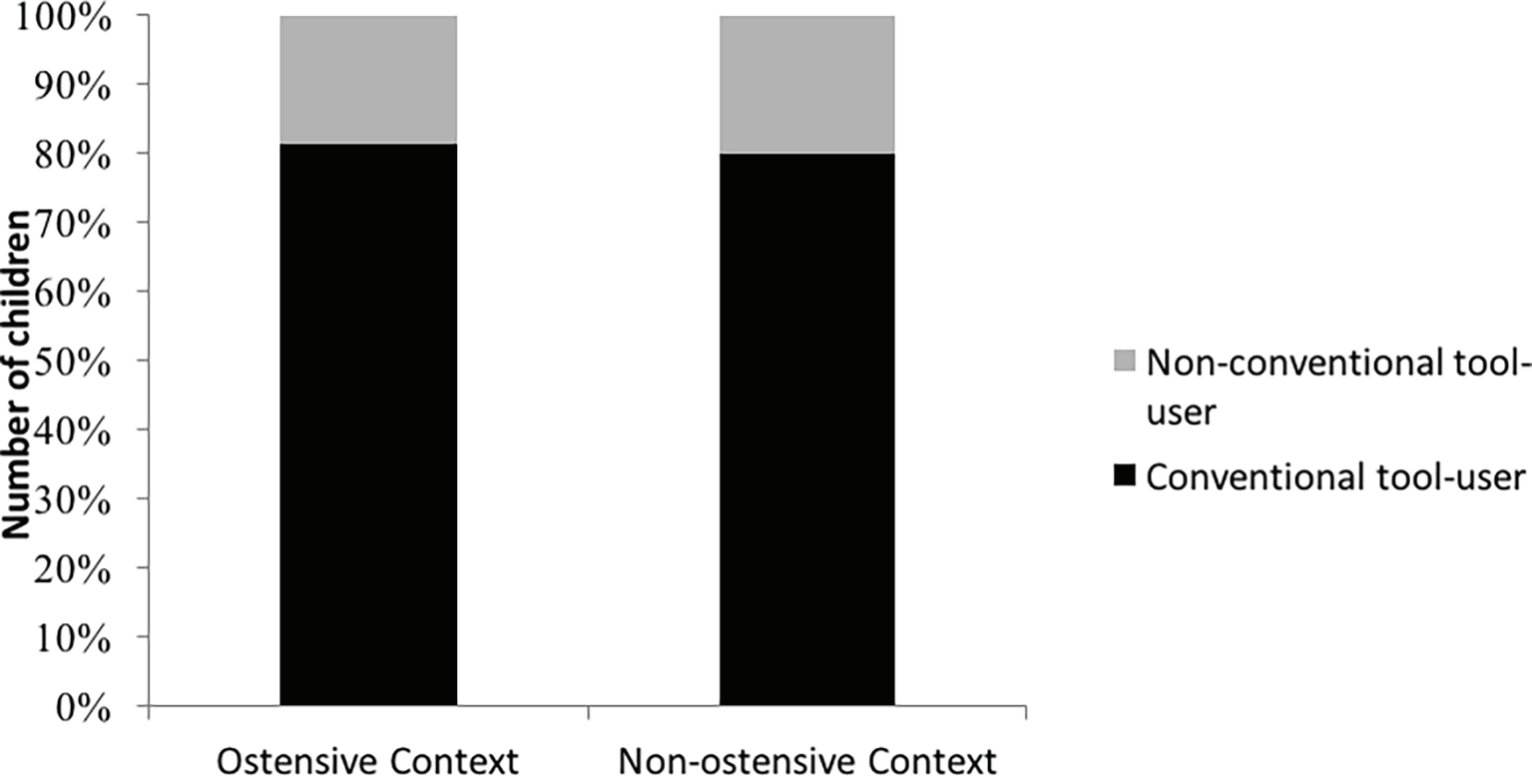
Number of children imitating the variants introduced by the two models in the ostensive and the non-ostensive conditions (excluding alternative solutions).

Due to the fact that they came up with a novel building method, 12 children were excluded from this analysis, leaving 17 children in the Non-ostensive and 16 children in the Ostensive condition. Similarly to the results of the first analysis, we found no difference in the distribution of behavior types between the conditions [*χ*^2^(1) = 0.113; *p* = 0.737], showing that the majority of children imitated the conventionally behaving model in both conditions (*n* = 13 in both conditions). A binomial test showed that children were altogether significantly more likely to perform the variant introduced by the conventionally behaving model (*p* = 0.001). The results are depicted in Figure 3.

## Discussion

This study tested whether 3-year-old children would selectively imitate a model whose competence in cultural knowledge was indicated by their tool-using habits. The results confirmed our hypothesis, showing that children were more willing to copy the behavior of someone whose behavior conformed to the cultural norms than that of someone who violated the culturally established norms. Oláh et al. (2014) provided evidence that the same behaviors that we used for familiarization in this study are associated with language use in children’s representations. Therefore, we propose that there may be a parallel in the selection mechanisms of children’s imitative behavior exhibited in our study and those showing selectivity based on linguistic cues (e.g., Buttelmann et al., 2013; Howard et al., 2015; Oláh et al., 2016). Both of these cues (language and conventionality in tool-using behavior) imply familiarity with the ways of a given culture; therefore, these selection mechanisms ensure that children endorse information and obtain practices that will likely be useful within their own environments.

The results also show that the selectivity based on the models’ prior behavior was not affected by the expression of communicative intentions in the test phase: children were just as unwilling to follow the behavior of an unconventionally behaving model in this case as they were when the models performed the actions in a non-communicative way. Thus, it seems that toddlers first identify the circle of reliable teachers and are reluctant to respond to the teaching intentions of those who fall outside of this circle. It is important to note that our study applied a forced choice method where children were always presented with a variant both from the conventionally and the unconventionally behaving model. Thus, it is possible that since children could not simply base their decisions about whom to follow on the perception of teaching intentions, they looked for other cues that could serve as guidance. We cannot be absolutely sure whether children would not imitate someone who does not keep to the cultural conventions but expresses their intentions to pass on knowledge if they are not presented with an alternative. However, similar studies on selective imitation of linguistic in-group members usually apply a between-subjects method and work with a communicative demonstration and also report reduced imitation rates of an out-group member (e.g., Howard et al. (2015), but see Buttelmann et al. (2013) for the same finding in a not particularly ostensive context). Thus, given the parallels between children’s reactions to linguistic out-group models and non-conformists to cultural norms, we would expect to see similar reluctance to imitate the latter following a communicative demonstration even when no alternative is presented. Note that in our design, there were no conditions that directly pitted ostension against conventionality, that is, where one model was conventional but produced no communicative signals, whereas the other behaved in a non-conventional way but expressed their willingness to teach. The reason for this choice was the fact that, based on previous findings, it would have been a viable prediction that both signals have equal significance in children’s eyes, and thus, they would choose at random (i.e., half of the children imitating one model, while the other half imitating the other model). This pattern of results would have been difficult to interpret as it could also be attributed to low-level cognitive mechanisms resulting in a random choice. Thus, it would have been impossible to differentiate between these two explanations.

It is possible that the lack of effect of ostension is related to the type of task children were presented with. Building blocks are familiar tools for children, and thus, the demonstration did not provide information about the function of a novel object, but rather about the preferences of the two models in how to use them. Although this may simply be viewed as an idiosyncratic behavior, another interpretation is that the different preferences arise from differences in “cultural practices.” We argue that the fact that children who decided to follow the behavior of either of the models chose the conventionally behaving one shows that they did not view the demonstrated action as idiosyncratic but as normative, at least within the given context. Note also that imitating actions with familiar tools may be a strong test of children’s motivation to align their behavior with another person or a group of people, since in such cases they do not have to acquire information about the basic affordances of the given tool.

However, the role of communication may be more pronounced when novices do not have prior information about the objects at hand. Yet, since the role of Natural Pedagogy in human cognition is to help foster the acquisition of opaque cultural knowledge (Csibra and Gergely, 2009), its effects could just as likely be manifested in relation to cultural practices (e.g., that we wave our hands when we want to say hello). It is worth noting that some studies with pre-schoolers have similarly found no effect of ostension on children’s behavior (e.g., Schmidt et al., 2011, with 3-year-olds and Hoehl et al., 2014, with 5-year-olds). We suggest that a viable explanation for such results is that children by the age of 3 are more sensitive to the wider social context of the testing and that simply the fact that the experimenter has drawn their attention to the demonstration videos nests the entire episode in a communicative context.

We believe it very likely that the relative importance of communicative cues and cues about cultural identity undergoes significant changes in the first years of life. It is possible that both the sensitivity to ostensive-referential signals (Csibra and Gergely, 2006) and the tendency to select teachers based on perceived group membership have innate roots; however, the latter is more strongly dependent on already stored information. Therefore, it may be adaptive for younger children to learn everything presented in a communicative context and later use the accumulated knowledge as anchors in subsequent learning episodes (see Whiten et al., 2005). Specifically, from these learning episodes, children can form expectations of how people should behave in certain situations, and violations of these expectations can prompt children to consider the reliability of the source of the information. It may also be an efficient strategy considering that the circle of people children meet in the first months of their lives is usually much more limited than in later years and is less likely to include people who may otherwise not be a part of the wider social group of children and would therefore communicate knowledge that is not valid for children. This idea is corroborated by findings with the overimitation method (presenting participants with an action sequence toward a specific goal where some elements of the sequence are clearly not causally necessary to arrive at the pre-set goal). Several researchers have shown that children have a tendency to copy even the obviously irrelevant elements of such action sequences (for a review see, Hoehl et al., 2019). Somewhat counterintuitively, the willingness to overimitate seems to increase with age. More specifically, preschoolers copy considerably more causally irrelevant actions than 2-year-olds (McGuigan and Whiten, 2009), while adults are even more faithful in their imitative behavior (McGuigan et al., 2011).

It has been suggested that humans’ tendency to overimitate is rooted in their motivation to comply with social norms and may accept arbitrary means toward a goal as the socially determined (i.e., conventional) way of doing things (e.g., Kenward et al., 2010; Herrmann et al., 2013; Keupp et al., 2013). Thus, it is possible that children’s developing social awareness and experience with social norms make them especially likely to perform actions simply to comply with these norms. If that is the case, children from around the age of 3 should be attentive to cues that suggest that the presented behavioral pattern may be relevant in their social environment or that they would be expected to conform to these behavioral patterns. Any cue about cultural knowledgeability of the source, including the tendency to use familiar tools in conventional ways, should foster these processes.

An important question that arises is whether the unconventional behaviors used in the familiarization phase in our study would lead children to form the impression that the person does not share cultural knowledge with themselves and consequently cannot be regarded as a member of the same cultural group. Children could have simply inferred that the person is “ignorant,” “funny,” or a “rule-breaker.” A number of studies have shown that children show selective learning based on similar behavior cues implying that the knowledge of the potential teacher is not reliable (e.g., Koenig and Harris, 2005; Pasquini et al., 2007). The study by Zmyj et al. (2010) applied a very similar method to ours where they introduced a model whose behavior deviated from the cultural norms and, importantly, who also signaled uncertainty about how to use the tools in front of him. In our study, the models always performed the actions with confidence in order to suggest that the person was not lacking knowledge, simply possessed *different* knowledge about the usage of the tools. Nonetheless, it is possible that children at this age do not differentiate between the two cases and treat both an uncertain model and a confident, but unconventionally behaving one as equally ignorant. Future research may address this question. However, even if children may not make the difference, the fact that they base their judgments about knowledgeability on conventionality of behavior is in itself informative. There may be other cues that could serve equally well as guidance about knowledgeability if the concept did not inherently include familiarity with cultural practices. For example, children could rely more strongly on cues of confidence or on the efficiency of the observed action. In our familiarization videos, the unconventional actions were always efficient in bringing about the highlighted goal. Children could also make the assumption that a person who finds a way to arrive at their goal is worth following; however, this was not the case: familiarity with the means to the goal played a crucial role. Moreover, mere familiarity was not sufficient to evoke trust as both the goal and the means were familiar in all the cases. “Unconventionality” was defined as the unexpected association of the two (otherwise familiar) elements of the actions; therefore beyond a sense of familiarity, top-down mechanisms sensitive to more subtle characteristics of the organization of behavior had to play a part. Thus, we suggest that for children (and adults as well), “knowledgeability” always includes familiarity with cultural practices. Therefore, even if children cannot explicitly postulate this, an unconventionally behaving person is not simply “ignorant” but not a good (conformist) member of a given social group as they do not share the established cultural knowledge.

To our knowledge, this study is the first one to show that language may not be the only relevant cue that provides grounds for selectivity in learning about cultural practices through signaling access to a specific body of cultural knowledge. We propose that tool-using habits and language both show children whether the interaction partner shares cultural knowledge with them and prefer to align their behavior with someone who seems knowledgeable in this respect. This selectivity ensures that children accumulate knowledge and behavioral patterns that are valid and useful in their social environment and filter out irrelevant pieces of information from the excess of stimuli reaching their cognitive system (e.g., meeting someone who speaks in a foreign language and performs a bow as greeting would not lead children to adopt this behavior when meeting someone else). We propose that the same sensitivity to known and unknown behavioral patterns (conformity to established practices) guiding children’s imitative behavior helps humans navigate a world filled with multiple dimensions of subcultures in adulthood as well.

## Data Availability

The dataset for this study can be found on the Open Science Framework website: https://osf.io/gcnfb/?view_only=c147d77438ae4c94af77a63ead63e176.

## Ethics Statement

Ethical approval for the study was obtained from the Research Ethics Committee of the Faculty of Education and Psychology of Eötvös Loránd University (Ref. no. 2014/127). Participants and parents signed informed consent prior to participation.

## Author Contributions

KO and IK designed the experiment. KO conducted the data collection and analyses. KO and IK wrote the paper.

### Conflict of Interest Statement

The authors declare that the research was conducted in the absence of any commercial or financial relationships that could be construed as a potential conflict of interest.

## References

[ref1] Boyd R. Richerson P. J. (1996). “Why culture is common, but cultural evolution is rare” in Proceedings-British Academy. Vol. 88 (Oxford: Oxford University Press Inc.), 77–94.

[ref2] Butler L. P. Markman E. M. (2012). Preschoolers use intentional and pedagogical cues to guide inductive inferences and exploration. Child Dev. 83, 1416–1428. 10.1111/j.1467-8624.2012.01775.x, PMID: 22540939

[ref3] Buttelmann D. Zmyj N. Daum M. Carpenter M. (2013). Selective imitation of ingroup over outgroup member in 14-month-old infants. Child Dev. 84, 422–428. 10.1111/j.1467-8624.2012.01860.x, PMID: 23006251

[ref4] Carpenter M. Akhtar N. Tomasello M. (1998). Fourteen-through 18-month-old infants differentially imitate intentional and accidental actions. Infant Behav. Dev. 21, 315–330. 10.1016/S0163-6383(98)90009-1

[ref5] Casler K. Kelemen D. (2005). Young children’s rapid learning about artifacts. Dev. Sci. 8, 472–480. 10.1111/j.1467-7687.2005.00438.x, PMID: 16246238

[ref6] Casler K. Kelemen D. (2007). Reasoning about artifacts at 24 months: the developing teleo-functional stance. Cognition 103, 120–130. 10.1016/j.cognition.2006.02.006, PMID: 16581053

[ref7] Casler K. Terziyan T. Greene K. (2009). Toddlers view artifact function normatively. Cogn. Dev. 24, 240–247. 10.1016/j.cogdev.2009.03.005

[ref8] Corriveau K. Harris P. L. (2009a). Preschoolers continue to trust a more accurate informant 1 week after exposure to accuracy information. Dev. Sci. 12, 188–193. 10.1111/j.1467-7687.2008.00763.x 19120427

[ref9] Corriveau K. Harris P. L. (2009b). Choosing your informant: weighing familiarity and recent accuracy. Dev. Sci. 12, 426–437. 10.1111/j.1467-7687.2008.00792.x0 19371367

[ref10] Csibra G. Gergely G. (2006). “Social learning and social cognition: the case for pedagogy” in Processes of change in brain and cognitive development. Attention and performance XXI, Vol. 21 (Oxford: Oxford University Press), 249–274.

[ref11] Csibra G. Gergely G. (2009). Natural pedagogy. Trends Cogn. Sci. 13, 148–153. 10.1016/j.tics.2009.01.005, PMID: 19285912

[ref12] Futó J. Téglás E. Csibra G. Gergely G. (2010). Communicative function demonstration induces kind-based artifact representation in preverbal infants. Cognition 117, 1–8. 10.1016/j.cognition.2010.06.003, PMID: 20605019

[ref13] Gergely G. Bekkering H. Király I. (2002). Developmental psychology: rational imitation in preverbal infants. Nature 415:755. 10.1038/415755a, PMID: 11845198

[ref14] Henderson A. M. Sabbagh M. A. Woodward A. L. (2013). Preschoolers’ selective learning is guided by the principle of relevance. Cognition 126, 246–257. 10.1016/j.cognition.2012.10.006, PMID: 23177705

[ref15] Henrich J. McElreath R. (2003). The evolution of cultural evolution. Evol. Anthropol. 12, 123–135. 10.1002/evan.10110

[ref500] Herrmann P. A. Legare C. H. Harris P. L. Whitehouse H. (2013). Stick to the script: The effect of witnessing multiple actors on children’s imitation. Cognition 129, 536–543. PMID: 24045001 10.1016/j.cognition.2013.08.010

[ref16] Hoehl S. Keupp S. Schleihauf H. McGuigan N. Buttelmann D. Whiten A. (2019). ‘Over-imitation’: a review and appraisal of a decade of research. Dev. Rev. 51, 90–108. 10.1016/j.dr.2018.12.002

[ref17] Hoehl S. Zettersten M. Schleihauf H. Grätz S. Pauen S. (2014). The role of social interaction and pedagogical cues for eliciting and reducing overimitation in preschoolers. J. Exp. Child Psychol. 122, 122–133. 10.1016/j.jecp.2013.12.012, PMID: 24569041

[ref18] Howard L. H. Henderson A. M. Carrazza C. Woodward A. L. (2015). Infants’ and young children’s imitation of linguistic ingroup informants. Child Dev. 86, 259–275. 10.1111/cdev.12299, PMID: 25263528 PMC4358791

[ref19] Jaswal V. K. Neely L. A. (2006). Adults don’t always know best preschoolers use past reliability over age when learning new words. Psychol. Sci. 17, 757–758. 10.1111/j.1467-9280.2006.01778.x 16984291

[ref20] Kelemen D. Carey S. (2007). “The essence of artifacts: developing the design stance” in Creations of the mind: Theories of artifacts and their representation (Oxford: Oxford University Press), 212–230.

[ref21] Kenward B. Karlsson M. Persson J. (2010). Over-imitation is better explained by norm learning than by distorted causal learning. Proc. R. Soc. B Biol. Sci. 278, 1239–1246. 10.1098/rspb.2010.1399 PMC3049065 20943697

[ref22] Keupp S. Behne T. Rakoczy H. (2013). Why do children overimitate? Normativity is crucial. J. Exp. Child Psychol. 116, 392–406. 10.1016/j.jecp.2013.07.002 23933292

[ref23] Király I. Csibra G. Gergely G. (2013). Beyond rational imitation: learning arbitrary means actions from communicative demonstrations. J. Exp. Child Psychol. 116, 471–486. 10.1016/j.jecp.2012.12.003, PMID: 23499323 PMC4636048

[ref24] Koenig M. A. Clément F. Harris P. L. (2004). Trust in testimony: children’s use of true and false statements. Psychol. Sci. 15, 694–698. 10.1111/j.0956-7976.2004.00742.x, PMID: 15447641

[ref25] Koenig M. A. Harris P. L. (2005). Preschoolers mistrust ignorant and inaccurate speakers. Child Dev. 76, 1261–1277. 10.1111/j.1467-8624.2005.00849.x, PMID: 16274439

[ref26] Legare C. H. Nielsen M. (2015). Imitation and innovation: the dual engines of cultural learning. Trends Cogn. Sci. 19, 688–699. 10.1016/j.tics.2015.08.005 26440121

[ref27] McGuigan N. Makinson J. Whiten A. (2011). From over-imitation to super-copying: adults imitate causally irrelevant aspects of tool use with higher fidelity than young children. Br. J. Psychol. 102, 1–18. 10.1348/000712610X493115, PMID: 21241282

[ref28] McGuigan N. Whiten A. (2009). Emulation and “overemulation” in the social learning of causally opaque versus causally transparent tool use by 23-and 30-month-olds. J. Exp. Child Psychol. 104, 367–381. 10.1016/j.jecp.2009.07.001, PMID: 19683722

[ref29] Nazzi T. Bertoncini J. Mehler J. (1998). Language discrimination by newborns: toward an understanding of the role of rhythm. J. Exp. Psychol. Hum. Percept. Perform. 24, 756–766. 10.1037/0096-1523.24.3.756, PMID: 9627414

[ref30] Oláh K. Elekes F. Bródy G. Király I. (2014). Social category formation is induced by cues of sharing knowledge in young children. PLoS One 9:e101680. 10.1371/journal.pone.0101680, PMID: 25014363 PMC4094464

[ref31] Oláh K. Elekes F. Pető R. Peres K. Király I. (2016). 3-year-old children selectively generalize object functions following a demonstration from a linguistic in-group member: evidence from the phenomenon of scale error. Front. Psychol. 7:963. 10.3389/fpsyg.2016.00963, PMID: 27445925 PMC4919341

[ref32] Pasquini E. S. Corriveau K. H. Koenig M. Harris P. L. (2007). Preschoolers monitor the relative accuracy of informants. Dev. Psychol. 43, 1216–1226. 10.1037/0012-1649.43.5.1216, PMID: 17723046

[ref33] Poulin-Dubois D. Brooker I. Polonia A. (2011). Infants prefer to imitate a reliable person. Infant Behav. Dev. 34, 303–309. 10.1016/j.infbeh.2011.01.006, PMID: 21353308

[ref34] Schmidt M. F. Rakoczy H. Tomasello M. (2011). Young children attribute normativity to novel actions without pedagogy or normative language. Dev. Sci. 14, 530–539. 10.1111/j.1467-7687.2010.01000.x, PMID: 21477192

[ref35] Senju A. Csibra G. (2008). Gaze following in human infants depends on communicative signals. Curr. Biol. 18, 668–671. 10.1016/j.cub.2008.03.059 18439827

[ref36] Southgate V. Chevallier C. Csibra G. (2009). Sensitivity to communicative relevance tells young children what to imitate. Dev. Sci. 12, 1013–1019. 10.1111/j.1467-7687.2009.00861.x, PMID: 19840055

[ref37] Topál J. Gergely G. Miklósi Á. Erdőhegyi Á. Csibra G. (2008). Infants’ perseverative search errors are induced by pragmatic misinterpretation. Science 321, 1831–1834. 10.1126/science.1161437, PMID: 18818358

[ref38] Vouloumanos A. Werker J. F. (2007). Listening to language at birth: evidence for a bias for speech in neonates. Dev. Sci. 10, 159–164. 10.1111/j.1467-7687.2007.00549.x, PMID: 17286838

[ref501] Whiten A. Horner V. Marshall-Pescini S. (2005). “Selective imitation in child and chimpanzee: a window on the construal of others’ actions” in Perspectives on Imitation: From cognitive neuroscience to social science. eds. Hurley S. Chater N. (MIT Press). PMID:

[ref39] Yoon J. M. Johnson M. H. Csibra G. (2008). Communication-induced memory biases in preverbal infants. Proc. Natl. Acad. Sci. USA 105, 13690–13695. 10.1073/pnas.0804388105 18757762 PMC2533251

[ref40] Zmyj N. Buttelmann D. Carpenter M. Daum M. M. (2010). The reliability of a model influences 14-month-olds’ imitation. J. Exp. Child Psychol. 106, 208–220. 10.1016/j.jecp.2010.03.002, PMID: 20427052

[ref41] Zmyj N. Daum M. M. Prinz W. Nielsen M. Aschersleben G. (2012). Fourteen-month-olds’ imitation of differently aged models. Infant Child Dev. 21, 250–266. 10.1002/icd.750

